# Whole-Genome Analysis of Influenza B Viruses of Multiple Genotypes Cocirculating in India

**DOI:** 10.1128/genomeA.00951-13

**Published:** 2013-11-27

**Authors:** Kunal N. Patil, Atul B. Bhise, Minal R. Dakhave, Archana A. Kadam, Mandeep S. Chadha, Varsha A. Potdar

**Affiliations:** National Institute of Virology, Pune, Maharashtra, India

## Abstract

Systematic influenza virus surveillance has been carried out in India since 2004 and has revealed the cocirculation of type B lineages. The genetic diversity of influenza B viruses was observed when full-genome analysis was performed. In 2010, the cocirculation of multiple genotypes was observed.

## GENOME ANNOUNCEMENT

The evolution of influenza B viruses has long been characterized by the cocirculation of antigenically and genetically distinct lineages for extended periods of time. Influenza B viruses of the B/Victoria/2/87 and the B/Yamagata/16/88 lineages have continued to cocirculate in many parts of the world since 1983 (1). Reassortants of the two lineages have been observed since 2002 (2) and have likely allowed unrestricted lineage mixing (3). A previous study of type B virus surveillance in India showed the cocirculation of the Victoria and Yamagata lineages (4).

The National Institute of Virology (NIV), Pune, India, as part of the WHO Global Influenza Surveillance Network, conducted virological and molecular surveillance of influenza viruses from different geographical regions of the country. In the present study, we carried out a whole-genome analysis of type B isolates from India to examine the reassortants and the evolutionary changes in type B lineages.

Type B influenza virus isolates grown in an MDCK cell line were studied for genetic characterization.

For whole-genome analysis, six isolates were selected. All eight influenza virus gene segments were amplified by one-step reverse transcriptase PCR (RT-PCR) using previously published primers (5). Sequencing was carried out using a BigDye Terminator v3.1 cycle sequencing ready reaction kit (ABI, USA), and samples were processed for capillary electrophoresis on an ABI 3730 DNA analyzer. Sequence alignment was done using MEGA (version 5.03), and phylogenetic trees were constructed using the neighbor-joining method based on Kimura’s 2-parameter distance matrix with 1,000 bootstrap replicates.

Phylogenetic analyses of the hemagglutinin (HA) gene of six type B isolates showed the clustering of three isolates each with Victoria-like (B/Brisbane/60/2008) and Yamagata-like (B/Florida/4/2006) strains. However, all isolates from the Victoria lineage were reassortants possessing the neuraminidase (NA) gene derived from the Yamagata lineage. All six isolates clustered with the Yamagata lineage in NA gene phylogeny. Within the Yamagata lineage, further subclustering of four isolates with B/Florida/4/2006 and two isolates with B/Brisbane/60/2008 (6) was observed. Whole-genome analysis of all six type B isolates showed grouping with either Victoria-like or Yamagata-like strains except for a nonstructural (NS) gene, which was grouped neither with Victoria-like nor Yamagata-like viruses. The isolates were assigned to clade A or B based on their tree topology when the NS gene was considered (7). Based on these groupings, different genomic patterns, namely, Vic+Vic+Yam+Vic+Yam+Yam+Yam+A/B for isolates NIV117519, NIV117528, and NIV1041860, Yam+Yam+Yam+Yam+Yam+Yam+Yam+A/B for isolates NIV117497 and NIV117504, and Yam+Vic+Yam+Yam+Yam+Yam+Yam+B for isolate NIV117501, were observed. The neuraminidase drug resistance markers E119A, R152K, D198N/E, I222T, H274Y, and R371K were not observed in the NA gene, and hence, all isolates remained oseltamivir sensitive. The continuous monitoring of influenza B viruses by whole-genome analysis is needed to understand the genetic diversity of influenza B viruses.

### Nucleotide sequence accession numbers.

The gene sequences of six Indian type B isolates from 2010 have been submitted to GenBank under the accession numbers listed in Table 1.

**Table 1:** List of accession numbers

Isolate	Accession no. for gene^*a*^:							
	PB1	PB2	PA	HA	NP	NA	M	NS
NIV117504	KF705471	KF705472	KF705473	KF705474	KF705475	KF364373	KF705476	KF705477
NIV117519	KF705478	KF705479	KF705480	KF705481	KF705482	KF364384	KF705483	KF705484
NIV117528	KF705485	KF705486	KF705487	KF705488	KF705489	KF364389	KF705490	KF705491
NIV117497	KF705492	KF705493	KF705494	KF705495	KF705496	KF364369	KF705497	KF705498
NIV117501	KF705499	KF705500	KF705501	KF705502	KF705503	KF314201	KF705504	KF705505
NIV1041860	KF705506	KF705507	KF705508	KF705509	KF705510	KF314368	KF705511	KF705512

a PB1, polymerase basic 1 gene; PB2, polymerase basic 2 gene; PA, polymerase acidic gene; HA, hemagglutinin gene; NP, nucleoprotein gene; NA, neuraminidase gene; M, matrix gene; NS, nonstructural gene.
